# Dissociable Posterior and Anterior Insula Activations in Processing Negative Stimulus Before and After the Application of Cognitive Reappraisals

**DOI:** 10.3389/fpsyg.2020.00268

**Published:** 2020-03-03

**Authors:** Ze Zhang, Tingting Guo, Jin Fan, Xiaofei Wu, Tengteng Tan, Jing Luo

**Affiliations:** ^1^Beijing Key Laboratory of Learning and Cognition, School of Psychology, Capital Normal University, Beijing, China; ^2^Key Laboratory of Mental Health, Institute of Psychology, Chinese Academy of Sciences, Beijing, China; ^3^Department of Psychology, Queens College, The City University of New York, New York, NY, United States

**Keywords:** insular cortex, cognitive reappraisal, creative cognitive reappraisals, emotion regulation, effectiveness

## Abstract

Although the role of the insular cortex in representing bodily and emotional feelings has been recognized, whether the mid-posterior and anterior parts of the insula act differentially in the encoding and regulation of emotional feelings is still unclear. In this functional magnetic resonance imaging (fMRI) study, we examined the effects of the creative cognitive reappraisals versus the non-creative ordinary reappraisals on the activation pattern of the mid-posterior and anterior insular cortex during the processing of unpleasant pictures by comparing the neural correlates for processing these pictures before and after the application of cognitive reappraisals. We found significant anterior insular activation after the application of cognitive reappraisals, especially the creative ones, in contrast to the significant mid-posterior insular activation before the application of the cognitive reappraisals or after the application of the non-creative ordinary reappraisals. This finding supports the posterior-to-anterior progression hypothesis with the mid-posterior insular cortex being used for the encoding of primary emotional feelings and the anterior insular cortex being used for the encoding of regulated or modulated emotional feelings.

## Introduction

Cognitive reappraisal, which is a cognitive and linguistic strategy for altering the trajectory of emotional responses by reformulating the meaning of a situation, has been widely studied and applied in emotion regulation (Gross and John, 2003). Although previous studies have identified multiple subcomponents of cognitive reappraisal, which mainly include the cognitive brain mechanisms for cognitive control (i.e., the lateral, ventral, and medial prefrontal cortex as well as the anterior cingulate cortex) and emotional arousal and feelings (i.e., the amygdala and insula) (Ochsner and Gross, 2008), the neural correlates of the changes produced by the reappraisal regulation in the bodily and emotional feelings, which could be theoretically regarded as one of the two essential components that constitute a given emotionality in addition to the component of motivations, induced by cognitive reappraisal are still unknown. There is converging evidence from the imaging of healthy (Critchley et al., 2004) and damaged brains (Wang et al., 2019) that demonstrates the critical role of the insular cortex in representing interoceptive attention and awareness of internal visceral responses, implying the insular cortex could be the key substrate for embodying the emotional feeling states. In particular, the insular activations have been widely observed during the application of emotion regulation strategies including both the explicit and implicit strategies (Diekhof et al., 2011; Langner et al., 2018; Picó-Pérez et al., 2019). More importantly, according to the posterior-to-anterior progression hypothesis on the hierarchical functional organization of the insular cortex (Craig, 2002, 2009), the posterior, middle, and anterior parts of the insula cortex are responsible for encoding primary bodily and emotional feelings, for mental re-representation of these feelings integrated with external context information, as well as for continuously updating introspective awareness of the present emotion and bodily states, respectively. Consistent with this theory, previous studies have demonstrated the double dissociation of activation in the mid-posterior and anterior insular cortex in the processing of sexual desire versus love (Cacioppo et al., 2012), primary versus secondary/modulated moral disgusts (Ying et al., 2018), and empathy and sympathy during childhood versus adulthood (with the latter showing increased frontalization and top–down modulation ability, Decety and Michalska, 2010).

The aforementioned posterior-to-anterior progression hypothesis (Craig, 2009) provides an ideal framework to formulize the neural encoding or representation of the changes in emotional feelings that accompany cognitive reappraisal applications. This is primarily because of the well-known role of the insular cortex in the representation of bodily and emotional feelings as well as the distinctive function of the posterior and anterior insula in the representation of the primary and the modulated bodily and emotional feelings. Prior to cognitive reappraisal application, it is possible that one’s initial representation of emotional feelings toward a negative stimulus might be relatively more intensively represented in the posterior insular cortex, which is responsible for the primary mental representation of emotional feelings. However, after application of cognitive reappraisal, the mid-posterior insular activation reflecting primary emotional feeling toward the negative stimulus might “move forward” to the anterior insula since the reinterpretation of the negative stimulus could result in a updated format of the emotional feeling representation with integration of the new meaning implied by reappraisal; in other words, after the application of emotion regulation, the anterior insular cortex may be more involved in the encoding or updating of the introspective awareness of emotional and bodily feeling states. In supporting of this possibility, on the one hand, previous studies have identified the role of the posterior insular cortex in encoding and regulating bodily and emotional feelings, including the aversive ones (e.g., Gehrlach et al., 2019), of both physical and social stimulus (e.g., Gao et al., 2018). On the other hand, the role of the anterior insular cortex in emotion regulation, including cognitive reappraisals, has also been consistently proved by previous studies (Diekhof et al., 2011; Langner et al., 2018; Picó-Pérez et al., 2019). Our recent study, which used a novel emotion regulation approach of “sadness counteract anger” (i.e., the pre-induced sadness emotion could reduce the later anger and aggressive behavior) and “fear promote anger” (i.e., the pre-induced fear emotion could enhance the later anger and aggressive behavior), found that the underlying mechanisms for the efficiency of this approach could be related to the effects of the pre-induced sadness could enhance the activation level in the posterior insular cortex (and thus enhance one’s internally orientated information processing tendency, which would reduce the externalized aggressive behavior), and the pre-induced fear emotion could enhance the activation level in the anterior insular cortex (and therefore facilitate an externalized angry expression), thus proving the possibility of making a double dissociation of the posterior and anterior insular cortex involvement during the application of different emotion modulation approaches (Zhan et al., 2018).

In order to explore how cognitive reappraisal could alter the emotional feelings embodied in the insular cortex, each trial of cognitive reappraisal of the target unpleasant picture consisted of the initial viewing of the target picture alone, the application of the cognitive reappraisal on the target picture, the re-watching of the target picture, and the final evaluation or appraisal of one’s subjective emotion. We examined our key prediction on the dissociation of the anterior and mid-posterior insular cortex by comparing the first- and the third-time passive viewing of the negative target pictures (i.e., before and after the application of reappraisals) and by comparing the consequences of different types of cognitive reappraisals.

We used three different types of reappraisals. In the objective description (OD) condition, the participants read sentences that just objectively described the contents of the negative pictures without any attempt at reappraisal regulation (baseline condition). In the ordinary reappraisal (OR) condition, the participants read reappraisal sentences that adopted the commonly used, non-creative reappraisal strategies, such as “they are getting help” (explicitly positive) or “the tragedy would never happen again” (changed future circumstances), to downregulate the elicited unpleasant emotion (McRae et al., 2012). In the creative reappraisal (CR) condition, the participants read reappraisal sentences that had an unexpected and insightful perspective that were both novel and appropriate and allowed for the reinterpretation of the meanings or implications of the pictures (for example, to reinterpret the vomit in toilet as a woman who wanted to have a baby surprisingly found herself to be pregnant).

The final type of creative cognitive reappraisal strategy was developed based on our recent study that found a significant correlation between the creativeness rating of the reappraisal and its efficiency (Wu et al., 2017). We therefore proposed a creative cognitive reappraisal theory (Wu et al., 2019) that suggested that the previously applied ordinary cognitive reappraisal strategies were too common or “mediocre” to efficiently overcome one’s emotional response bias, which is deeply rooted in one’s information-processing tendency toward negative situations. This could resemble the situation in creative insight problem solving where one’s mental fixation on the old way of thinking could prevent one from finding novel and suitable solutions. We therefore suggested that truly creative and insightful cognitive reappraisal could greatly improve the unfavorable mental representation toward negative stimuli. Consistent with this hypothesis, our recent study found that creative or insightful reappraisal could produce a regulation effect size (Cohen’s d, a measure of the effectiveness of emotion regulation) of 3.49, which was significantly higher than that reported in previous meta-analyses on cognitive reappraisal (all less than 0.95) (Augustine and Hemenover, 2009; Webb et al., 2012). Moreover, our previous study showed that the creative or insightful cognitive reappraisal resulted in an emotional rating that was above the neutral emotion level for standardized International Affective Picture System (IAPS) negative pictures (this means the CRs was able to turn the negative pictures to be perceived as positive) and that the application of this strategy was associated with robust positive activation in the amygdala, hippocampus, and ventral striatum, implying this change could be mediated by the enhanced activation in the reward circuits (Wu et al., 2019).

In this study, we compared the involvement of the mid-posterior and anterior insular cortex in the processing of standardized negative IAPS stimuli before and after the application of different types of cognitive reappraisal with different emotion regulation effectiveness. We predicted that cognitive reappraisal would alter the participants’ emotional feelings, which are essentially represented and re-represented in the insular cortex, in a manner that was consistent with the posterior-to-anterior progression hypothesis of this area (Craig, 2009) and that this tendency would be associated with the effectiveness of the emotion regulation strategy. By this we mean the CR strategy, which was found to be more effective in downregulating the negative emotion arousal associated with the processing of unpleasant IAPS pictures, would be more capable in making such a posterior-to-anterior insular activation progression than the ORs and the objective descriptions (CD) conditions, which were found to be less effective in doing so.

## Materials and Methods

It should be noted that some of the results of this study have been published (Wu et al., 2019); however, this paper reports the results of different experimental data. As previously mentioned, each experimental trial of cognitive reappraisal of the negative IAPS pictures consisted of four information-processing steps: the first-time picture viewing (Step 1), the application of reappraisal on the target pictures (Step 2), the second-time picture viewing (Step 3), and the final emotion rating (Step 4). In our previous study, we analyzed and reported the imaging data in Step 2 (Wu et al., 2019). In this study, we compared the insular activation in Step 1 (before reappraisal) and 3 (after reappraisal) to test our key hypothesis. We have previously reported information on the participants, materials, and experimental procedure (Wu et al., 2019). Here, we briefly introduce these details.

### Materials

The functional magnetic resonance imaging (fMRI) experiment used 75 negative IAPS pictures depicting the threat and attack scenario as well as disgust things and animals. Their mean valence rating was 2.56 (*SD* = 0.52), and their mean arousal rating was 5.43 (*SD* = 0.85), both on a nine-point scale. Given the difficulty in generating genuine insightful reappraisals on one’s own (Wu et al., 2017), we made a list of ordinary and creative insightful reappraisal sentences for the negative IAPS pictures in advance and presented them to the participants to induce creative or ordinary reappraisal. Although studying the process of passively reading and comprehending cognitive reappraisals provided by the experimenter was not as ideal as investigating the process of the participants generating cognitive reappraisals by themselves, this triggering approach provided a reasonable solution to the difficulty of studying the neural correlates of insightful restructuring. By this we mean the participants’ self-generation of the highly creative cognitive reappraisals are especially hard, if not impossible, and this made it difficulty to meet the technical requirement of an event-related fMRI, which needs a sufficient number of target mental events with accurate onset time (Luo and Knoblich, 2007), if we adopted a self-generated reappraisal paradigm.

**FIGURE 1:**
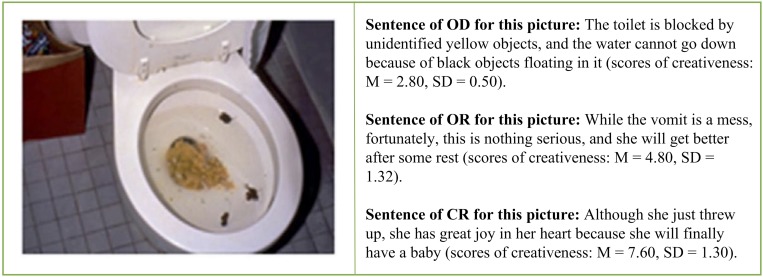
Example of three types of reappraisal.

We prepared three types of reappraisal interpretations for each of the 75 negative pictures; namely, objective description (OD), ordinary reappraisal (OR), and the creative reappraisal (CR). We obtained the CR sentences from the pool of 947 CR items toward the 75 negative IAPS pictures we mentioned earlier (Wu et al., 2017). The mean creativeness score (the extent to which the participant felt that the reappraisals were novel and unexpected) was 6.56 (*SD* = 1.57), the effectiveness score (the extent to which the description could improve one’s emotional feelings) was 6.46 (*SD* = 1.71), and the appropriateness score (the extent to which the description was fit for the scene depicted in the picture) was 5.79 (*SD* = 1.85). Figure 1 shows examples of the three types of reappraisals.

### Participants

Thirty-one college students (17 females; age: 24 ± 2.01, range: 21–27) participated in this study as paid volunteers. We excluded data from eight participants due to technical or cognitive reasons (see Wu et al., 2019, for details). The experimental protocol was approved by the Institutional Review Board of the Institute of Biophysics, Chinese Academy of Sciences. Written informed consent was obtained from all participants before the experiment.

### Procedure

The whole experimental procedure involved five experimental phases that included various types of follow-up evaluations and ratings performed 10 min or 3 days after the first phase (Wu et al., 2019). In the present paper, we have only reported the first phase that involved MRI scanning directly related to the goal of this study. In this phase, each of the 75 pictures and the corresponding reappraisal sentence were presented one by one in a random order. Regarding the exact procedure for each trial, the participants were first shown the target picture for 2 s and were required to passively view it (Step 1). Next, after a 1- to 3-s unfilled delay, the picture was presented together with an OD/OR/CR sentence for 12 s (Step 2). The participants were required to attentively read these sentences and attempt to understand their meanings and implications for the target picture (this 12-s duration has been focally analyzed and reported in our previous study, Wu et al., 2019). Next, after a 2- to 6-s unfilled delay, the target picture was presented again without any reappraisals for 2 s (Step 3), and the participants were again required to passively view it, which was also followed by a 1- to 3-s unfilled delay. Finally, the participants were required to indicate their emotional valence associated with processing the target picture using two emotionality-rating tasks (Step 4, see Wu et al., 2019 for details of this rating procedure).

For each participant, the 75 pictures were randomly assigned to one of the three conditions (OD, OR, and CR) with 25 trials in each condition. For each given picture, each participant could only see one type of the OD, OR, or CR sentences to avoid possible interactions among different types of reappraisals for the same picture. Valence and arousal were not significantly different in the three conditions (*p*_*all*_ > 0.05). Prior to the start of the formal fMRI scanning experimental session, the participants received thorough instructions and were sufficiently trained using another set of similar materials with an identical procedure.

### Image Acquisition and Preprocessing

We performed fMRI on a Siemens 3T Trio MRI scanner (Siemens Medical Systems, Erlangen, Germany). Functional scans were acquired using T2^∗^-weighted gradient echo, echo-planar pulse sequences. The following acquisition parameters were used in the fMRI protocol: TR = 2,000 ms, TE = 30 ms, slice number = 32, flip angle = 90-degree, matrix size = 64 × 64, FOV = 220 mm × 220 mm, and voxel size = 3.4 mm × 3.4 mm × 3 mm. For each participant, functional data were acquired in three scanning sessions containing 416 vol per session. The total lasted for 2,496 s (1,248 scans, TR = 2), which separated into three equal-length scanning sessions. Stimulus were presented on an MR-compatible monitor using E-prime software (Psychology Software Tools). Participants were in a supine position with their heads snugly fixed by a belt and foam pads to minimize head motion.

Functional MRI data was subjected to preprocessing steps using SPM8 software package (Statistical Parametric Mapping, Wellcome Department of Cognitive Neurology, London, United Kingdom): slice timing, realigned to the first volume, co-registered to the T1 image, normalized to a standard template [Montreal Neurological Institute (MNI)], resampled to a spatial resolution of 3 mm × 3 mm × 3 mm, spatially smoothed with an 8 mm × 8 mm × 8 mm full-width-at-half-maximum (FWHM) Gaussian kernel and 128s high-pass filtering (0.011 Hz).

### Functional MRI Data Analysis

A functional MRI data analysis was conducted using SPM8. For the first level analysis, general linear modeling (GLM) was conducted for the functional scans from each participant. Several regressors were created by convolving a train of delta functions, which consisted of a synthetic hemodynamic response function (HRF): the first-time passively viewing of three types of target pictures, which would be subsequently re-presented together with the corresponding CR, OR, or OD sentences respectively in Step 1 (defined as the event of CR1, OR1, and OD1); the processing of the three kinds of reappraisal materials (CR reappraisal sentence processing, OR reappraisal sentence processing, and OD description sentence processing, defined as the event of CR2, OR2, and OD2) in Step 2; as well as the second-time passively viewing of the three kinds of target pictures, which had just been paired with CR, OR, or OD sentences (defined as event of CR3, OR3, and OD3, respectively) in the Step 3. All events were entered as parametric regressors at the onset of picture display. Moreover, two picture rating events were also entered as out of interest regressors. Six parameters generated during motion correction were entered as covariates. Linear contrasts of the parameter estimates were made to identify the effects of the nine picture viewing events for each participant. These images from all participants were then entered into a second-level group analysis conducted with a random-effects statistical model with the threshold of *p* < 0.001 (uncorrected) and 50 or more continuous voxels (K ≥ 50).

Regions of interest (ROIs) in the insular cortex were defined using structural AAL templates for the left and right insular cortex in WFU_PickAtlas_3.0.3^1^, which was used as an inclusive mask and overlapped with the functionally activated clusters based on certain contrasts (e.g., the contrast of “CR1 minus CR3” or that of “CR3 minus OR3”). Given the insular activation in processing unpleasant pictures were theoretically predicted by related theories and research results on emotional arousal and regulation, we took a relatively loose threshold, *p* < 0.05 (uncorrected), to get a comprehensive examination on possible insular activation changes associated with the application of different types of reappraisal approach. In addition to that, we extracted the percent signal changes in the bilateral posterior and anterior insular cortex based on a given contrast using the MarsBar^2^. We selected the contrasts, which we defined the insular ROIs in such a way that the contrast was independent of the targets contrast we were interested in. The percent signal changes within these ROIs were extracted for each condition separately for each participant and were further averaged across all participants to produce mean scores.

To investigate the relationship between insula activations and subjective ratings of pleasantness, we also correlated each participant’s percent signal change within the given functional ROIs with subjective rating of pleasantness for the CR condition.

## Results

The behavioral results showed that pictures paired with CR were rated as more pleasant than those paired with OR and OD. In addition, pictures paired with OR were rated as more pleasant than those paired with OD, implying that the emotional regulatory effectiveness of the three conditions was CR > OR > OD (Wu et al., 2019).

**FIGURE 2:**
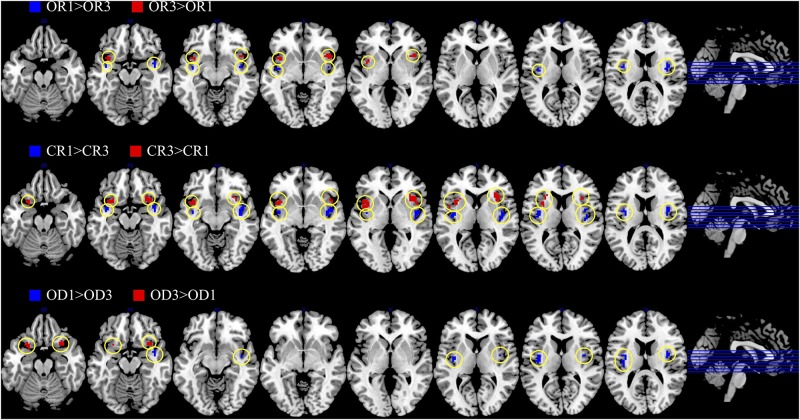
The anterior and posterior insular activations observed before and after the application of different types of cognitive reappraisals (OR or CR) and objective descriptions (OD).

**FIGURE 3:**
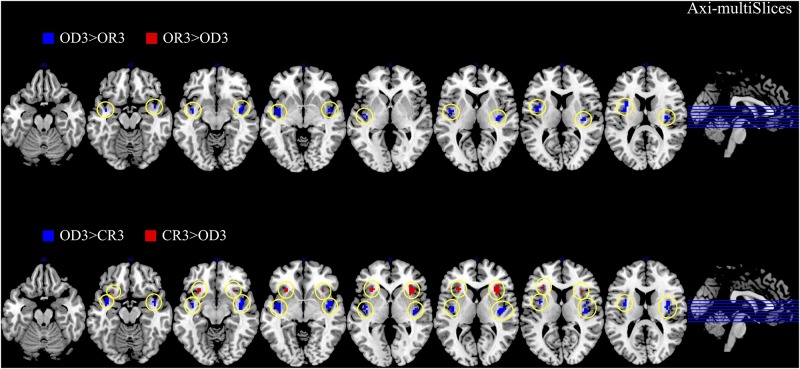
The anterior and posterior insular activations observed after the application of creative reappraisals (CR) and objective descriptions (OD) (transverse plane).

**FIGURE 4:**
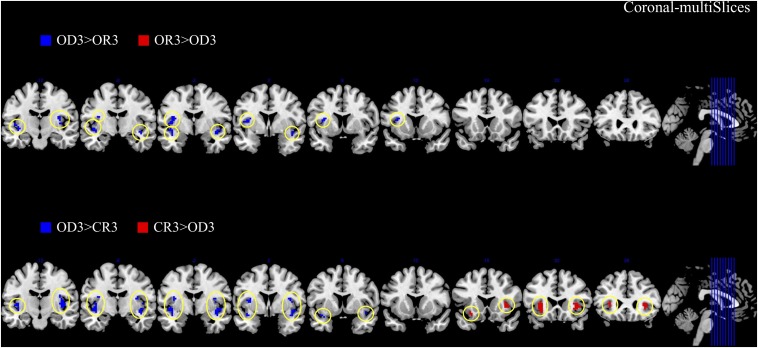
The anterior and posterior insular activations observed after the application of creative reappraisals (CR) and objective descriptions (OD) (coronal plane).

**FIGURE 5:**
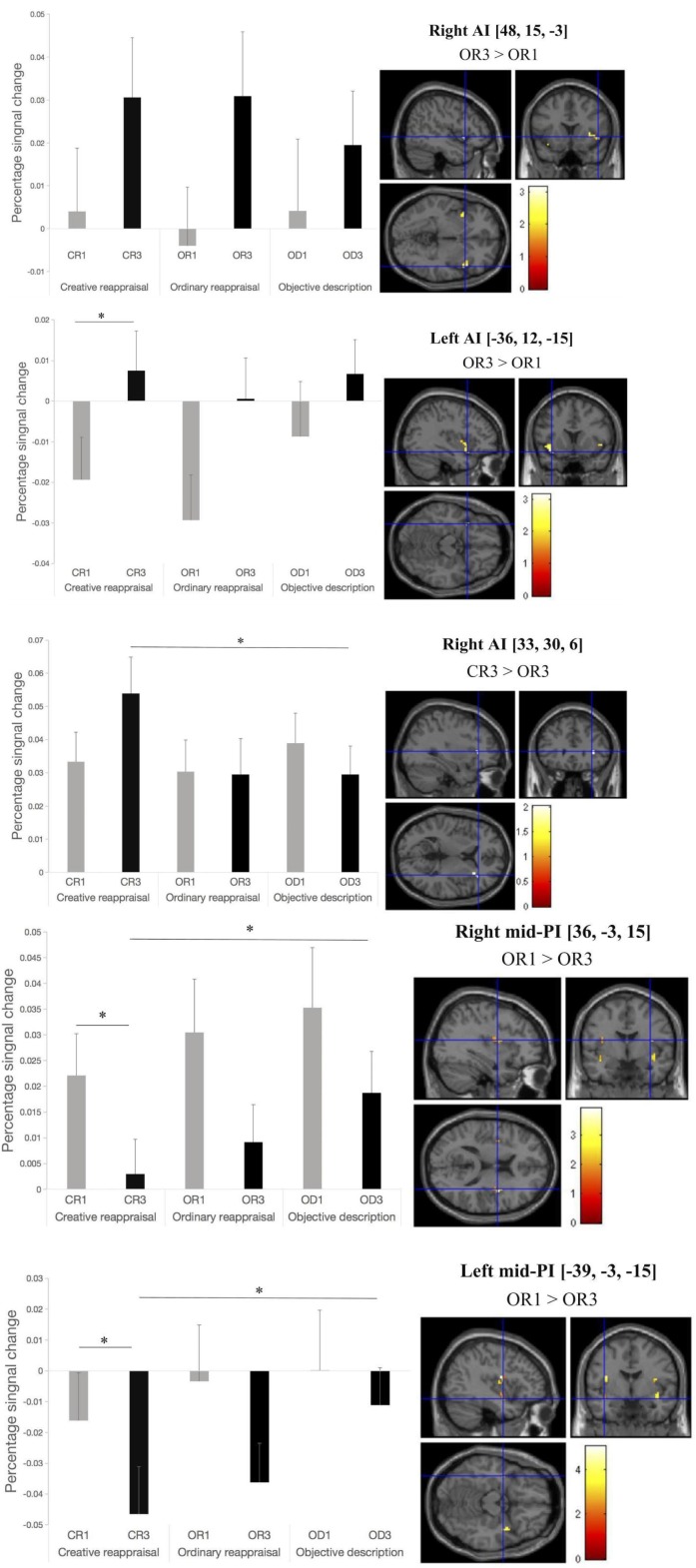
Activation in mid-posterior and anterior insular ROIs before and after the application of cognitive reappraisal or objective descriptions. Note that all the differences that reached a significant level were marked as ^∗^ (*p* < 0.05) or ^∗∗^ (*p* < 0.001) except for the contrast based on which we defined the ROIs. The location of the peak voxel in the given ROI and the contrast based on which we defined the ROIs were provided on the right top of each picture.

The brain imaging results found more anterior insular activations after the application of cognitive reappraisals or objective descriptions relative to the one before (OR3 > OR1, CR3 > CR1, OD3 > OD1, Figures 2, 5 and Table 1) as well as after the application of creative or ordinary reappraisals relative to that of objective descriptions (OR3 > OD3, CR3 > OD3, Figures 3–5 and Table 1). In contrast, more posterior insular activations were found before the application of reappraisals relative to the one after (OR1 > OR3, CR1 > CR3, OD1 > OD3, Figures 2, 5 and Table 1) as well as after the application of objective descriptions relative to that of creative or ordinary reappraisals (OD3 > OR3, OD3 > CR3, Figures 3–5 and Table 1). Additionally, we also provided the results of a whole brain analysis with the threshold of *p* < 0.001 (ucr), K ≥ 50 in Table 2, but no super-threshold insular activation was observed at this threshold.

**TABLE 1:** MNI coordinates of the peak insular activation observed in three contrasts between Step 1 and Step 3 for each condition (i.e., “CR 1 vs. CR3,” “OR 1 vs. OR3,” and “OD 1 vs. OD3”) and three contrasts across condition in Step 3 (i.e., “CR 3 vs. OD3,” “OR 3 vs. OD3,” and “CR3 vs. OR3”).

**Brain regions**	**MNI coordinates**			**T**	**K**
	**x**	**y**	**z**		
**CR3 > CR1**					
L anterior insula	−24	12	−18	3.155	119
R anterior insula	48	15	−3	3.153	59
R anterior insula	33	18	−15	2.779	30
**CR1 > CR3**					
L mid-posterior insula	−36	−6	18	5.090	61
R mid-posterior insula	42	3	−15	4.070	109
**OR3 > OR1**					
L anterior insula	−36	12	−15	2.589	31
R anterior insula	48	15	−3	2.485	31
**OR1 > OR3**					
R mid-posterior insula	42	0	−15	3.234	15
R mid-posterior insula	36	−3	15	2.809	14
L mid-posterior insula	−39	−3	15	2.651	18
L mid-posterior insula	−39	−3	−15	2.041	11
**OD3 > OD1**					
L anterior insula	−24	15	−21	3.880	15
R anterior insula	27	15	−18	3.199	22
**OD1 > OD3**					
L mid-posterior insula	−39	−9	12	4.209	52
R mid-posterior insula	42	0	−15	3.271	14
R mid-posterior insula	39	−3	15	2.617	13
**CR3 > OD3**					
R anterior insula	33	24	6	4.283	56
L anterior insula	−27	21	−3	2.847	41
**OD3 > CR3**					
L mid-posterior insula	−42	−9	−3	3.928	62
R mid-posterior insula	39	0	15	3.301	120
L mid-posterior insula	−33	0	15	2.856	23
**OD3 > OR3**					
L mid-posterior insula	−39	6	12	4.072	84
R mid-posterior insula	36	−15	6	2.823	37
R mid-posterior insula	45	0	−9	2.714	24
OR3 > OD3	No suprathreshold clusters				
CR3 > OR3	No suprathreshold clusters				
OR3 > CR3	No suprathreshold clusters				

*(1) Threshold of voxel levels: T = 1.717, *p* < 0.05 (unc.), K = 10, inclusive mask = structural insula; MNI = Montreal Neurological Institute.*

**TABLE 2:** MNI coordinates of peak activation associated with three contrasts between Step 1 and Step 3 for each condition (i.e., “CR1 vs. CR3,” “OR1 vs. OR3,” and “OD1 vs. OD3”) and three contrasts across condition in Step 3 (i.e., “CR3 vs. OD3,” “OR3 vs. OD3,” and “CR3 vs. OR3”).

	**MNI coordinates**			**T**	**K**
**Brain regions**	**x**	**y**	**z**		
**CR3 > CR1**					
R Precuneus	6	−78	48	10.037	1492
R Middle frontal gyrus	42	33	36	9.585	1169
R Inferior parietal lobule	51	−42	45	8.640	783
R Caudate	21	−39	9	8.512	432
R Pyramis	45	−78	−33	7.413	445
L Middle frontal gyrus	−36	45	6	7.250	729
L Inferior parietal lobule	−48	−63	51	6.865	505
L Inferior temporal gyrus	−48	−3	−39	6.777	307
R Middle temporal gyrus	42	3	−39	6.192	52
R Inferior temporal gyrus	60	−39	−15	6.191	321
R Lentiform nucleus	15	12	−12	5.717	88
L Precentral gyrus	−54	12	6	5.217	50
L Medial frontal gyrus	−15	9	−15	5.181	65
R Precentral gyrus	54	12	9	4.778	65
**CR1 > CR3**					
L Inferior occipital gyrus	−39	−81	−6	14.980	5312
R Middle frontal gyrus	42	12	30	9.314	428
L Middle frontal gyrus	−39	−3	48	6.585	327
L Cerebellar tonsil	0	−51	−42	6.012	104
R Inferior frontal gyrus	48	30	9	5.834	56
**OR3 > OR1**					
R Precuneus	6	−75	48	8.448	1603
L Inferior parietal lobule	−57	−48	51	7.714	637
R Middle frontal gyrus	36	54	6	7.519	891
R Inferior parietal lobule	45	−45	42	7.237	676
R Middle temporal gyrus	45	6	−42	7.133	71
L Middle frontal gyrus	−21	45	−12	6.107	517
R Middle temporal gyrus	63	−21	−12	5.456	183
L Middle temporal gyrus	−45	3	−42	5.418	82
L Medial frontal gyrus	−15	9	−18	5.061	51
R Medial frontal gyrus	12	12	−15	4.881	56
L Middle frontal gyrus	−33	27	42	4.717	96
**OR1 > OR3**					
R Middle occipital gyrus	39	−87	6	13.733	5551
R Middle temporal gyrus	51	0	−21	6.684	62
R Inferior frontal gyrus	42	9	30	6.295	270
L Precentral gyrus	−39	−6	48	6.071	95
L Inferior frontal gyrus	−42	6	30	6.059	126
L Middle frontal gyrus	−36	33	−15	5.665	59
L Inferior semi-lunar lobule	−9	−78	−39	5.327	51
**OD3 > OD1**					
R Precuneus	12	−72	36	9.508	4707
R Middle temporal gyrus	45	6	−42	6.936	67
R Tuber	42	−81	−30	6.495	121
R Inferior parietal lobule	48	−42	39	6.374	577
L Inferior parietal lobule	−60	−45	48	6.169	342
R Superior frontal gyrus	24	27	51	4.742	124
Left cerebellum inferior semi-lunar lobule	−21	−87	−39	4.628	56
**OD1 > OD3**					
L Inferior occipital gyrus	−33	−84	−9	14.158	5055
L Precentral gyrus	−36	−6	51	7.320	430
R Inferior frontal gyrus	42	12	27	7.208	532
R Middle temporal gyrus	51	−6	−18	6.705	85
L Tonsil	0	−51	−42	6.429	76
L Inferior semi-lunar lobule	−9	−78	−39	5.747	119
**CR3 > OD3**					
Right cerebellum	15	−45	−24	6.253	139
L Precentral gyrus	−33	−24	57	5.812	83
R Middle frontal gyrus	33	57	6	4.975	75
L Superior frontal gyrus	−36	15	51	4.542	56
R Declive	12	−72	−9	4.235	61
**OD3 > CR3**					
R Precentral gyrus	39	−21	54	6.575	390
L Medial frontal gyrus	−3	60	0	5.947	75
**OD3 > OR3**	No suprathreshold clusters				
**OR3 > OD3**	No suprathreshold clusters				
**CR3 > OR3**	No suprathreshold clusters				
**OR3 > CR3**	No suprathreshold clusters				

*Threshold of voxel levels: T = 3.505, *p* < 0.001 (uncorrected), K ≥ 50 voxels.*

To further address our main hypotheses about the dissociable effects of the posterior and anterior insular cortex, we conducted two kinds of repeated-measures analyses of variance (ANOVAs) on the percent signal changes in insular cortex as *post hoc* analysis. One type was 3 (reappraisal type: OD, OR, CR) × 2 (regulation phase: before and after reappraisal) repeated measure ANOVAs. The other was 2 (insula area: anterior insula [AI] and mid-posterior insula [PI]) × 3 (reappraisal type: OD, OR, and CR) × 2 (regulation phase: before and after reappraisal) repeated measure ANOVAs. The former 3 × 2 ANOVAs took the percent signal changes in the given insular ROIs as the function of the factors of the reappraisal type and regulation phase, whereas the latter 2 × 3 × 2 ANOVAs took the averaged percent signal changes in several left or right AI and PI ROIs as the function of the factors of the insula area, reappraisal type, and regulation phase.

The results of the former 3 (OD, OR, CR) × 2 (before and after reappraisal) ANOVAs are summarized in Table 3. As is shown in Table 3, in some insular RIOs, we found the significant main effects of the reappraisal type and/or reappraisal phase.

**TABLE 3:** Summary of the anterior and posterior insular ROIs that demonstrated the main effects of regulation phases and/or reappraisal types in the 3 (reappraisal type: OD, OR, and CR) x 2 (regulation phase: before and after) repeated measure ANOVAs.

			**Results of 3 (reappraisal type: OD, OR, and CR) x 2**	
			**(regulation phase: before and after) repeated measures ANOVA**	
			
**Regions**	**Contrasts based on which the ROIs were defined**	**Location of peak voxels in ROI [x,y,z]**	**Main effects of regulation phases (before vs. after emotion regulation)**	**Main effects of reappraisal types (OD vs. OR vs. CR)**
Bilateral anterior insula (AI)	OR3 > OR1	Right AI [48,15,−3]	*F*_(__1_,_22__)_ = 5.838, *p* = 0.024, *$\eta_{\text{p}}^{2}$* = 0.210; SEA: CR3 > CR1 (*p* = 0.062).	
		Left AI [−36,12,−15]	*F*_(__1_,_22__)_ = 8.470, *p* = 0.008, *$\eta_{\text{p}}^{2}$* = 0.278; SEA: CR3 > CR1 (*p* = 0.001).	
	CR3 > OR3	Right AI [33,30,6]	*F*_(__1_,_22__)_ = 3.521, *p* = 0.038, $\eta_{\text{p}}^{2}$ = 0.138; SEA: CR3 > CR1 (*p* = 0.073).	*F*_(__2_,_44__)_ = 4.124, *p* = 0.023, *$\eta_{\text{p}}^{2}$* = 0.158; SEA: CR3 > OD3 (*p* = 0.011).
Bilateral mid-posterior insula (mid-PI)	OR1 > OR3	Right mid-PI [36,−3,15]	*F*_(__1_,_22__)_ = 7.568, *p* = 0.012, *$\eta_{\text{p}}^{2}$* = 0.256; SEA: CR3 < CR1 (*p* = 0.011).	*F*_(__2_,_44__)_ = 3,744, *p* = 0.034, *$\eta_{\text{p}}^{2}$* = 0.145; SEA: OD3 > CR3 (*p* = 0.036)
		Left mid-PI [−39,−3,−15]	*F*_(__1_,_22__)_ = 4.943, *p* = 0.037, *$\eta_{\text{p}}^{2}$* = 0.183; SEA: CR3 < CR1 (*p* = 0.024).	*F*_(__2_,_44__)_ = 5.303, *p* = 0.009, *$\eta_{\text{p}}^{2}$* = 0.194; SEA: OD3 > CR3 (*p* = 0.011).
		Right mid-PI [42,0,−15]	*F*_(__1_,_22__)_ = 18.575, *p* < 0.001, *$\eta_{\text{p}}^{2}$* = 0.458; SEA: CR3 < CR1 (*p* = 0.001), OD3 < OD1 (*p* = 0.046).	*F*_(__2_,_44__)_ = 5.145, *p* = 0.012, *$\eta_{\text{p}}^{2}$* = 0.190.
		Left mid-PI [−39,−3,15]	*F*_(__1_,_22__)_ = 13.430, *p* = 0.001, $\eta_{\text{p}}^{2}$ = 0.379; SEA: CR3 < CR1 (*p* = 0.003), OD3 < OD1 (*p* = 0.007).	*F*_(__2_,_44__)_ = 4.390, *p* = 0.018, *$\eta_{\text{p}}^{2}$* = 0.166.

*SEA: simple effect analyses using Bonferroni correction.*

The 2 (AI, PI) × 3 (OD, OR, CR) × 2 (before and after reappraisal) ANOVAs were conducted on the mean percent signal change in the right lateral AI and PI regions (including the right AI ROIs that peaked at [x,y,z = 48,15,−3] and [33,30,6] as well as the right PI ROIs that peaked at [x,y,z = 36, −3,15] and [x,y,z = 42,0,−15]) and the left lateral AI and PI regions (including the left AI ROIs that peaked at [x,y,z = −36,12,-15] as well as the left PI ROIs that peaked at [x,y,z = −39, −3,15] and [x,y,z = −39,−3,−15]). The analysis results are reported below, starting with main effects and then two-way and three-way interactions.

For the right insular ROIs, we found significant main effects of the insula area (AI vs. PI), *F*_(__1_,_22__)_ = 5.703, *p* = 0.026, *$\eta_{\text{p}}^{2}$* = 0.206, but no significant main effects of regulation phase (before vs. After reappraisal), *F*_(__1_,_22__)_ = 0.814, *p* = 0.377, *$\eta_{\text{p}}^{2}$* = 0.036 and reappraisal type (CR vs. OR vs. OD), *F*_(__1_,_22__)_ = 1.144, *p* = 0.328, $\eta_{\text{p}}^{2}$ = 0.049, indicating that the right AI ROIs exhibited greater activation than the right PI ROIs across different reappraisal types and regulation phases.

Analysis of two way interactions revealed significant interaction between insula area and regulation phase, *F*_(__1_,_22__)_ = 18.708, *p* < 0.001, $\eta_{\text{p}}^{2}$ = 0.46, and insula area and reappraisal type, *F*_(__2_,_44__)_ = 10.641, *p* < 0.001, $\eta_{\text{p}}^{2}$ = 0.326, but no significant interaction between reappraisal type and regulation phase, *F*_(__2_,_44__)_ = 0.141, *p* = 0.869, $\eta_{\text{p}}^{2}$ = 0.006. More critically, we found a significant three-way interaction of insula area, regulation phase, and reappraisal type, *F*_(__2_,_44__)_ = 3.778, *p* = 0.031, $\eta_{\text{p}}^{2}$ = 0.147, indicating that, before regulation, the percent signal change of all regulation types (CR1, OR1, and OD1) in both AI and PI ROIs had no significant differences. However, a simple effect analysis using the Bonferroni correction indicated that the percent signal change after the application of CR in the right PI ROIs was significant lower than OD (i.e., CR3 < OD3, *p* = 0.043; OR3 vs. OD3 and CR3 vs. OR3, *p_*all*_* > 0.05), but no significant differences were found between reappraisal types in the right AI ROIs (CR3 vs. OD3, OR3 vs. OD3, and CR3 vs. OR3, *p_*all*_* > 0.05). Likewise, for the left insula, we also found a significant three-way interaction of insula area, regulation phase, and reappraisal type, *F*_(__2_,_44__)_ = 3.696, *p* = 0.033, $\eta_{\text{p}}^{2}$ = 0.144, indicating that, before regulation, the percent signal change of all reappraisal type (CR1, OR1, and OD1) in both AI and PI ROIs had no significant differences. However, simple effect analysis using the Bonferroni correction indicated that the percent signal change after the application of CR in the right PI ROIs was marginally significant lower than OD (i.e., CR3 < OD3, *p* = 0.058; OR3 vs. OD3 and CR3 vs. OR3, *p_*all*_* > 0.05), but no significant differences were found between regulation type in the right AI ROIs (CR3 vs. OD3, OR3 vs. OD3, and CR3 vs. OR3, *p_*all*_* > 0.05).

We correlated each participant’s percent signal change within the functional ROIs with subjective rating of pleasantness for the CR condition. However, no significant correlations were found between insula activation and the pleasantness ratings.

## Discussion

Given that the functions of the mid-posterior and insular cortex can be segregated, with the mid-posterior insular cortex playing a greater role in representing physiological reactivity and homeostatic states, and the anterior insular cortex playing a greater role in integrating homeostatic afferent activity from the dorsal posterior insula with emotional salience to form a global representation of the bodily feeling state (Craig, 2002, 2009), we made a key hypothesis in the present study that cognitive reappraisal would alter the emotional feelings (toward negative stimulus) represented in the insular cortex in a posterior-to-anterior progression manner (Craig, 2009). Consistent with this hypothesis, we found that application of the cognitive reappraisals, especially the creative ones, could induce increased anterior insular activation and reduced mid-posterior insular activation.

Increased mid-posterior insular activations were found when the first-time viewing of the unpleasant pictures (in Step 1) was contrasted with the third-time viewing (in Step 3) (i.e., CR1 > CR3) or across the conditions of different regulation strategies (e.g., OD3 > CR3). The mid-posterior insular cortex was more activated before the application of reappraisal regulation or in the OD condition relative to CR condition, and this could be related to the fact that participants had more intensive unpleasant emotional feelings before the reappraisal regulation or after the application of cognitive reappraisals in OD condition relative to CR condition. In support of this possibility, previous studies have generally demonstrated the function of the posterior insular cortex in encoding disgust sensory and emotional feelings (Gehrlach et al., 2019), such as unpleasant haptic sensations (Bonenberger et al., 2015), heat pain (Koenen et al., 2017), cold temperatures (King and Carnahan, 2019), and even some kinds of social emotionality like disgust (Britton et al., 2006; Ying et al., 2018).

In contrast, increased anterior insular activations were found when the third-time viewing (in Step 3) was contrasted with the first-time viewing of the unpleasant pictures (in Step 1) (i.e., CR3 > CR1) or the after the application of CRs relative to that of ODs. The anterior insular cortex activation has been widely observed in the processes of emotion regulation, and this includes both of the explicit regulations—the cognitive reappraisal (Morawetz et al., 2017) and expressive suppression (Giuliani et al., 2011)—as well as the implicit ones, such as the fear extinction and placebo (Diekhof et al., 2011; Picó-Pérez et al., 2019); this implies that the role of anterior insular cortex in emotion regulation could be either conscious and effortful (in explicit emotion regulation) or automatic and effortless (in implicit emotion regulation), which was in some sense inconsistent with the general impression that the anterior insular cortex, together with ACC, makes critical contributions to the function of cognitive control (Schmeichel et al., 2008; Gyurak et al., 2012). Regarding the function of the anterior insular cortex, a more fundamental possibility is it simply serves as the mechanism for making the mental representation or appraisal of the emotional responses or emotional arousal rather than for regulating emotion (Fullana et al., 2018; Picó-Pérez et al., 2019). This raises the question of what kind of function the anterior insular cortex actually served in the application of cognitive reappraisals in the present study. Did it simply mean the mental representation or emotional appraisal of the negative emotionality after watching unpleasant pictures? Or did it mean the regulation of that negative emotional arousal? And, if it did mean some regulation-related processing, did it embody the “processes” of emotion regulation or the “results” of it?

Firstly, we think our observation of anterior insular cortex activation in this study could be more related to the emotion regulation process rather than to the encoding or appraisal of the negative pictures because the anterior insular cortex was more activated after the application of reappraisals than before, and it was more activated after the application of CRs relative to that of ODs. If the “negative emotion appraisal account” was true, we might find more anterior insular cortex activation in the reverse contrasts. Secondly, we think the anterior insular cortex activation observed in the present could be more related to the “results” or “consequences” of the emotion regulation rather than the “process” of emotion regulation because the “process” of emotion regulation should had been mainly accomplished in the Step 2 of the experiment (the presentation of reappraisals, which was not the focus of the present study, Wu et al., 2019). Despite this, we still could not completely exclude the possibility that participants continued to make the emotion regulation when they saw the picture again, especially in the case of CR, which could take more intensive information processing to integrate the implication of reappraisal sentences with the scenes depicted by the unpleasant pictures. Thirdly, based on the first and second points of consideration, a further question we may ask is, if the anterior insular cortex activation we observed in the present study did embody—or embodied to a large extent—the “results” rather than the “process” of the emotion regulation; did it represent the encoding or appraisal of the emotional arousal toward the unpleasant pictures itself or to the newly established emotionality induced by the reinterpretation of cognitive reappraisals? To this question, two sides of considerations may help us to make a guess: (a) previous studies have proved the cognitive reappraisal exerted its emotional regulatory effects through an “indirect” approach that altered the semantic representation toward the unpleasant event (this could be mainly accomplished by the cognitive control regions together with the lateral temporal cortex) and finally changed the property of the emotional arousal embodied in emotional regions, such as the amygdala (Buhle et al., 2014), thus implying the importance and the involvement of “the mental representational change” in the application of the reappraisal strategy; and (b) our fMRI study on CRs found robust and positive reward circuit activations, including the ones in ventral striatum, amygdala, and hippocampus, during the application of creative cognitive reappraisal (relative to that of the ordinary and OD) in Step 2 (Wu et al., 2019), thus implying the importance and the involvement of “the positive mental representational change” (in at least the application of CRs). These two considerations reminded us that the activation of anterior insular cortex observed in the present study could not only be related to the encoding or appraisal of the emotional arousal toward the unpleasant pictures itself; rather, it could reflect the emotional appraisal of the newly established emotionality that integrated the content of cognitive reappraisals with the target pictures. This possibility was also consistent with, for example, Taylor and colleague’s study that found the anterior insula was co-activated with the anterior cingulate cortex (ACC) to form an essential part of the salience network, which detected salient events and initiated appropriate control signals to regulate behavior and the homeostatic state (Taylor et al., 2009). It was also generally consistent with the findings of previous neuroimaging studies, which suggested that the anterior insular cortex was involved in cognitive control (Wager and Feldman Barrett, 2004; Ochsner and Gross, 2008; Aziz-Zadeh et al., 2009; McCrea, 2010) given the obvious need for executive functions, such as working memory capacity, inhibition, and category shift in emotion regulation, and the fact that the cognitive reappraisals have to be sustained against competing bias (Schmeichel et al., 2008; Gyurak et al., 2012).

In summary, we compared the involvement of the posterior and anterior insular cortex in processing negative stimuli before and after cognitive reappraisal regulation and found that reappraisal could alter the internal bodily and emotional feeling representation in the insular cortex in a posterior-to-anterior progression manner. The more powerful an emotion regulation strategy was (e.g., in the case of CR condition relative to the OR and CD conditions), the more obvious posterior-to-anterior insular activation progression tendency the strategy would be able to make. Our study indicates a clear dissociation of the functions of the posterior and anterior insular cortex associated with the application of reappraisal strategies, which demonstrates the change of the internal bodily and emotional feeling representation patterns produced by the reappraisal strategy. This provides new evidence toward the systematic and comprehensive understanding of the cognitive brain mechanisms through which the cognitive reappraisal exerts its emotional feeling regulation effects.

The limitation of this study includes (a) the experimental design of passively viewing of pre-prepared cognitive reappraisal materials undermined the “ecological validity” of the study, and this approach was relatively rarely adopted in previous studies on cognitive reappraisals and it could also rarely occur in the real world psychotherapy practice; (b) we did not find any significant correlation between the activation of the insular cortex and the participants’ subjective evaluation or appraisal of their emotional feelings, and this implied the activation in the insular cortex may just embody some elements of one’s emotional feelings, and these elements could not directly impact the holistic subjective evaluation of emotions; and (c) we observed insular activation at a lenient threshold (*p* < 0.05, uncorrected). One possible reason for this was that all the six experimental conditions we compared were just the passive viewing of the same set of unpleasant IAPS pictures (before and after the application of three different kinds of reappraisals). This relatively minor between-condition difference might have made it difficult to obtain robust insular activation. However, given the function of insular cortex in processing unpleasant stimulus and that on participating cognitive reappraisals were well-documented by previous studies, we think the results of the present study were meaningful and reflected the dissociable function of the mid-posterior and the anterior parts of insula in representing the regulation effects on one’s emotional feeling by reappraisal strategy. Further studies using more ecologically valid designs (such as have participants to make the cognitive reappraisals on their own) and more sensitive technologies or approaches to detect the information processing signals in insular cortex and to detect one’s subjective emotional feelings, as well as their changes, are needed to further clarify these limitations.

## Data Availability Statement

All datasets generated for this study are included in the article/supplementary material.

## Ethics Statement

The studies involving human participants were reviewed and approved by the Institutional Review Board of the Institute of Biophysics, Chinese Academy of Sciences. The patients/participants provided their written informed consent to participate in this study. Written informed consent was obtained from the individual(s) for the publication of any potentially identifiable images or data included in this manuscript.

## Author Contributions

TG and JL designed and supervised the study. TG collected the data. TT, ZZ, and XW analyzed the data. ZZ, JL, JF, and TT wrote the manuscript.

## Conflict of Interest

The authors declare that the research was conducted in the absence of any commercial or financial relationships that could be construed as a potential conflict of interest.

## Footnotes

1 http://fmri.wfubmc.edu/software/PickAtlas

2 http://marsbar.sourceforge.net
